# Changing priorities in the development of cognitive competence and school learning: A general theory

**DOI:** 10.3389/fpsyg.2022.954971

**Published:** 2022-09-29

**Authors:** Andreas Demetriou, George Charilaos Spanoudis, Samuel Greiff, Nikolaos Makris, Rita Panaoura, Smaragda Kazi

**Affiliations:** ^1^Cyprus Academy of Sciences, Letters, and Arts, Nicosia, Cyprus; ^2^University of Cyprus, Nicosia, Cyprus; ^3^University of Luxembourg, Luxembourg, Luxembourg; ^4^Democritus University of Thrace, Komotini, Greece; ^5^Frederick University, Nicosia, Cyprus; ^6^Panteion University, Athens, Greece

**Keywords:** educational priorities, developmental priorities, theories of cognitive development, school achievement, learning deficiencies

## Abstract

This paper summarizes a theory of cognitive development and elaborates on its educational implications. The theory postulates that development occurs in cycles along multiple fronts. Cognitive competence in each cycle comprises a different profile of executive, inferential, and awareness processes, reflecting changes in developmental priorities in each cycle. Changes reflect varying needs in representing, understanding, and interacting with the world. Interaction control dominates episodic representation in infancy; attention control and perceptual awareness dominate in realistic representations in preschool; inferential control and awareness dominate rule-based representation in primary school; truth and validity control and precise self-evaluation dominate in principle-based thought in adolescence. We demonstrate that the best predictors of school learning in each cycle are the cycle’s cognitive priorities. Also learning in different domains, e.g., language and mathematics, depends on an interaction between the general cognitive processes dominating in each cycle and the state of the representational systems associated with each domain. When a representational system is deficient, specific learning difficulties may emerge, e.g., dyslexia and dyscalculia. We also discuss the educational implications for evaluation and learning at school.

## Introduction

This article presents a comprehensive theory which deals with the relations between cognitive development and school learning. This theory aims to enable education to enhance cognitive development and improve the attainment of learning goals associated with each school year. Schools capitalize on cognitive competence to enable students to acquire, through the years, new skills and concepts ranging from literacy and numeracy skills to complex concepts and skills in science, mathematics, and the arts. Anything at school is demanding at the beginning. School classrooms are complex environments. Children in each classroom differ in competence, interest to learn, personality, and family background. Teachers differ in education, teaching skills and styles, and persistence to drive students to learn. School subjects differ in their conceptual characteristics and demands; for instance, learning language requires a different combination of cognitive processes than learning mathematics or science. These differences make it admirable that students do learn at school. Unfortunately, however, students often fail to learn what education plans in every school subject at each grade.

The theory proposed here aspires to bridge cognitive developmental priorities with educational priorities from preschool to adolescence. We have two aims: first, to strengthen cognitive development of individual students at each school year to fully reach their potential; unfulfilled developmental priorities at successive school years often deprive learners from learning skills and knowledge needed to master the cognitive abilities associated with a specific year to efficiently move forward. Second, we also aim to build on the possibilities afforded by developmental priorities at successive school grades to maximize learning in each school subject. We claim that ignoring these priorities may cause difficulties and delays in grasping and consolidating the concepts and skills of interest (Demetriou and Spanoudis, 2018). We focus on general problem-solving processes and problem solving in two domains: language and mathematics. Table 1 summarizes developmental priorities, educational priorities and learning goals in problem-solving, language, and mathematics across age periods and domains, and, finally, learning difficulties associated with each phase.

**Table 1:** Developmental priorities, educational priorities, and learning across developmental cycles.

Age/cycle	Developmental priorities	Symbol systems	Problem-solving priorities	Language priorities	Priorities in mathematics	Learning difficulties
Early Preschool 2–3 years	Attention control.	Mastering language and understanding the role of symbols.	Explore action-based solutions.	Command basic grammatical and syntactical rules.	Magnitude representation. Early number sense.	Failure in inducing basic rules in different domains.
Late Preschool 4–5 years	Perceptual awareness.	Building connections between symbol systems: e.g., photographs picture real objects.	Learn to build a representation for a problem, plan, evaluate, e.g., sorting, trial-and-error exploration of causality.	Acquire basic phonological awareness; pre-writing activities.	Map number words on number digits, relevant images of objects. Counting. Grasp cardinality and quantitative comparisons.	Difficulties in attention control and coordination between a represented goal and action.
Early Primary 6–9 years	Inferential control, Rule induction, inductive reasoning.	Symbol and representational integration. Symbols are mental objects that can be combined and operated on, such as number digits.	Fill in lags in information by inference. Constructions following a plan, e.g., Lego constructions.	Learn how letters, syllables, and words stand for speech sequences. Combine words into meaningful sentences. Writing.	Explicit grasp of the mental number line. Exploration of the patterns between one-digit numbers, tens, and decades to grasp rules underlying structure of place value system.	Dyslexia, Dyscalculia. Difficulties in inducing or applying rules for the management of representations.
Late Primary 9–12 years	Inferential awareness.	Understanding that symbol systems are exchangeable. Words, mental images, pictures, and numbers may denote the same realities.	Specify when available knowledge or solutions are not enough and look for or invent new ones: e.g., simulate worlds as in Minecraft.	Abstract different lines of meaning in texts. Evaluate rationale underlying text, even when text covers multiple themes so that main idea can only grasped if analyzed at several levels.	Control rules specifying numeric relations and patterns and transform them to each other. Recognition of relations between numbers must extent from integers to rational numbers, complex numbers.	Crystallization of mental processes and knowledge into ready-to-draw on mental skills and concepts.
Early secondary 13–15 years	Truth and consistency control, Principles, deductive reasoning.	Learning of abstract symbolic systems, such as algebra.	Think for possible solutions and then reject bad solutions, choose promising ones according to criteria. Isolation of variables.	Fluent reading and story production in writing. Understand abstract ideas in various texts, e.g., newspapers, scientific texts, literature.	Grasp algebraic number as a variable that may take any value and that operations on numbers depends on their precise nature	Difficulties in forming a personal symbol system for handling and manipulating abstract concepts
Late Secondary 16–18 years	Awareness of constraints in reasoning, accurate self-representation, and self-evaluation.	Grasp of the special role of scientific notation as language systems standing for models of reality.	Be creative and original, choosing best solutions even against personal biases or believes. State hypotheses and test accordingly.	Read texts in different domains, mastering domain-specific special language. Connect ideas across domains.	Modeling complex situations using mathematics; specify similarities and differences between problem situations; compute diverse types of fractions and decimals.	Difficulties in grasping metatheoretical/ epistemological assumptions within and across knowledge domains.

Education looked for direction and teaching practices in psychological theories since the early 20th century. The interaction between education and psychology met with both successes and failures. Here, we do not discuss in detail psychological theories which have been important for education in the past. However, to embed the present theory in context, we attempt a brief overview of the main ideas drawn from them and evaluate their contribution to the development of education of our time. We then present a comprehensive model drawing on the successes and failures of the past and capitalizing on recent research.

The ideas presented in this paper developed over a long time. The empirical basis of the developmental priority model appeared in a series of empirical studies mapping the developmental priorities of successive developmental cycles (Demetriou et al., 2017, 2018a, 2022a; Makris et al., 2017; Kazi et al., 2019). The educationally relevant studies appeared in another series of empirical papers which explored how learning happens in different domains and how cognitive developmental profiles are related to school performance at different school levels (Demetriou et al., 2019a, 2020a,b, 2021, 2022b). The theoretical and educational implications appeared in several papers (Demetriou, 2020a; Demetriou et al., 2020b, 2021, 2022b).

This paper synthesizes this research into a comprehensive framework used here to account for learning in two important domains of school life, language, and mathematics, and explain related learning difficulties. We first review cognitive training research to show that this research fell short of expectations to increase cognitive ability because it ignored changes in developmental priorities. We then focus on the present model, following the organization of themes in Table 1, first summarizing the basic postulates and findings of the theory, and then focusing on their educational implementation.

## Taking stocks of promising models that did not fulfill all promises

### Research aiming to boost intelligence and cognitive processes

Psychology of individual differences systematically influenced education since early 20th century. Binet and Simon (1916) developed the first intelligence test in France to identify children facing difficulties to learn (Anastasi and Urbina, 1997). The tests that followed proved successful in satisfying this aim. Later, several programs aimed to boost the intelligence of children diagnosed by these tests to face learning difficulties at school. These programs targeted processes addressed by intelligence tests, such as reasoning and knowledge in different domains. The Head Start Program in the United States is a major endeavour planned to improve the learning skills of poor children (Neisser et al., 1996; Neisser, 1998). A similar program, the Abesedarian study, focused on children at risk. Training involved activities and problem solving in various domains of mathematics and language (Campbell and Burchinal, 2008). The outcome of this research is easy to summarize. The students involved gained up to 7–8 IQ points but the end of the training program. However, this effect faded out soon after the end of the program; in 3 years after intervention gains dropped to meager 1–2 points (Protzko, 2015, 2016).

Why are gains from intelligence training so fragile? Pessimists claim that general intelligence is a stable trait impervious to interventions. Interventions affect superficial skills, such as test-taking skills, which degrade soon after intervention ends (Jensen, 1998; Murray, 2020). The environmentalist view assumes that interventions may change intelligence, but gains are sustainable only if the benevolent environment remains present (Ceci, 1991; Ceci and Williams, 1997). The developmental explanation espoused here ascribes the fade out effect to the fact that the studies designed to increase intelligence were developmentally insensitive, ignoring that the nature of intelligence changes with development. Boosting a specific form of a cognitive ability that is important at a given age does not ensure that it will transfer to other forms which are important in later phases.

In recent years, learning research focused on general-purpose cognitive processes associated with psychometric general intelligence, g (Carroll, 1993; Jensen, 1998): namely, processing efficiency, executive functions, and working memory. Scholars assumed that boosting each of these processes would increase general intelligence (e.g., Jaeggi et al., 2008; Protzko, 2015). Evidence is clear: these processes do improve because of training but gains *do not transfer* to g, everyday problem-solving, or academic achievement (Shipstead et al., 2012, 2016; Melby-Lervåg et al., 2016; Sala and Gobet, 2017, 2020).

These studies ignored the direction of causality in the relations between these processes and general intelligence and their role in development. Specifically, hierarchical models of the structure of intelligence, such as the three-stratum model proposed by Carroll (1993), assume that causality goes top-down, from g to each component process, rather than bottom-up, from component processes to g (Protzko, 2015, 2016). Therefore, transfer of learning relies on the training of core markers of general intelligence shared by the component processes rather than the component processes as such. Training specific processes recruited by general intelligence, when needed, such as attention or working memory, would not improve general intelligence, if core processes would remain unchanged. Change would occur only if training central processes used to regulate inhibition or working memory, such as relational or awareness processes. Also, the role of each process in general intelligence and learning differs at successive developmental phases, according to their role in satisfying the cognitive developmental priorities in each phase (Demetriou et al., 2013, 2014, 2020a, 2021).

### Research capitalizing on developmental theories

The theories of Piaget (1970), Vygotsy (1986), and Bruner (1973) influenced education significantly in the second half of 20th century. These theories raised awareness that children of different ages understand the world differently from adults and choose distinct aspects of an episode to attend; hence, their view of the world must be respected by education, if efficient learning is to occur. This implies recognizing developmental constraints of learning: if concepts and skills are to be learned by children, they must be presented at the level of abstraction and complexity that is appropriate for their developmental level. Eventually, however, the popularity of these theories declined because they failed to improve day-to-day teaching and learning at school. There were reasons for their decline. First, these theories were too global to direct the formation of educational priorities and programs at the level of detail needed by education for day-to-day teaching or even successive school grades. For instance, in mathematics, children need to learn, grade by grade, a vast array of concepts and skills, such as understanding and operating with diverse types of number (e.g., integers, decimals, fractions, and algebra). Learning each of these concepts and skills often requires specific representational and integrative processes. These were ignored by general theories.

Second, these theories underestimated the complexity of development, emphasizing some processes more than other processes. The theory of Piaget (1970) defined cognitive development as a linear progression towards an ideal end state of logical reasoning, formal operations. As a result, it reduced cognitive development to the development of reasoning, and downplayed other processes, such as self-regulation and awareness. Thus, Piaget’s theory failed to explain difficulties of learning in school related to processes other than reasoning. For instance, primary school children do not know how to adjust their learning according to tasks (Annevirta and Vauras, 2001; Digmath et al., 2008). Thus, memorizing material may fall short of the effort required because students think, wrongly, that what is in front of their eyes now it would be available in memory later. Therefore, stressing reasoning is not enough to ensure the learning of the concepts involved.

### Research capitalizing on conceptual change

The conceptual change approach to learning and cognitive development emerged in the eighties in reaction to the weakness of the theories above to account for understanding of specific concepts (Carey, 1985; Vosniadou, 2013). In a way, this approach turned priorities upside down, emphasizing domain-specificity and downplaying general processes. According to this approach, children construct models about the world as they interact with various phenomena, such as physical, astronomical, or biological phenomena. For instance, they have a model explaining the day-night cycle on earth. Children revise models in the fashion scientists revise scientific theories. Theories survive in as long as they serve their purpose to explain incoming information in a domain; if current information deviates from expectations, theories are revised.

Learning in this context is based on several principles: Children must “do” science actively–observe, experiment, test hypotheses, compare observations with their beliefs, reflect, and revise, if evidence does not accord with expectations. Teachers must take children’s mental models seriously and build environments for them where they can express their concepts about a phenomenon, manipulate objects according to their expectations, and compare findings with expectations and expert models, and revise them accordingly. For instance, to understand the day-night cycle, children must replace their model assuming that the sun is orbiting around earth by the model assuming that earth rotates around its axis. However, research suggests that students often do not learn the scientific concepts we teach them. Wrong models and misconceptions persist, even among specialists, because restructuring dominant concepts in favor of scientific concepts requires processing and inferential possibilities exceeding students’ current developmental capabilities (King, 2010).

## Fundamental principles for tuning education with developmental priorities

The research reviewed above suggests that enhancing intelligence is possible, but gains are not sustainable over time. Training specific processes is also possible, but gains are not transferable to the core of intelligence. Letting children be active and constructive when they learn is useful, but learning is weak if not properly directed to the cognitive processes that would allow capitalize on the cognitive processes enabling understanding in each phase. Failure of learning experiments to transfer to real life must be contrasted to the broad success of formal schooling to raise abilities. Each extra year of schooling enhances cognitive development (Kyriakides and Luyten, 2009) and raises intelligence by 2–4 IQ points (Gustafsson, 2008; Ritchie and Tucker-Drob, 2018). Also, increases in general intelligence in the population, known as the Flynn effect, which amount to about 25 IQ points over the 20th century, are related to the expansion of formal education throughout the 20th century (Flynn, 2009).

The model of developmental priorities presented here suggests that this is due to three aspects of schooling: First, schooling is long lasting, recurrent, and repetitive. It extends from early childhood to early adulthood and concepts and skills often recycle over different grades, increasingly complexified, expressed in different contexts and different symbol systems. Second, it addresses both general and specific mechanisms in the context of different subjects, promoting reflection, bridging of concepts, abstraction, and reconceptualization. Third, even if not very systematically, it familiarizes children with the use of different symbol or representation systems, such as mathematical, linguistic, visual, or musical notation. This helps to decontextualize mental representations and transformation from its actual contents and facilitates mental flexibility in moving across systems.

Therefore, a major challenge for any theory of education is to distill what is most important in school from each of these sources, refine it, and give it back better targeted and programmed. The aim is a balanced model integrating the three traditions outlined above (psychometric, cognitive, and developmental research on the human mind). In this model, the constructive approach to learning must specify the nature of cognitive competence at different periods of development; use developmentally sensitive methods of scaffolding learning at successive school grades throughout school life, capitalizing on developmental priorities and possibilities of successive phases of life. Teachers must teach concepts at the representational resolution, inferential power, abstraction refinement, and flexibility in conceptual revision that is possible at different developmental periods (Carey, 1985, 2009; Siegler and Jenkins, 1989).

## Cognitive architecture, development, and school learning

The present theory aligns cognitive developmental priorities with educational priorities. Satisfying cognitive developmental needs at successive developmental phases would maximize the possibilities of individuals to master learning tasks associated with successive levels in education. This theory is based on the principles of cognitive architecture and development summarized below.

### Cognitive architecture

The architecture of the human mind includes the following constructs.

#### A general factor

Research in the psychology of individual differences strongly suggests that *a powerful factor of general cognitive ability constrains learning and understanding.* Through the years, this factor has come under various names. For psychology of individual differences, it is general intelligence or g. Widely used tests of intelligence, such as the WISC or the Raven test, are reliable measures of this factor (Spearman, 1904; Carroll, 1993; Jensen, 1998). This factor stands for the processes following:

1. Align and inter-relate incoming information of interest according to a represented goal guiding search of information in the environment.
2. Abstract patterns and make connections by inference, if information is missing, and conclude if they fit or deviate from what was known or believed so far.
3. Make choices, interpretations, or decisions best serving current priorities or interests.
4. Capitalize on feedback from past choices, decisions, or actions, to improve the predictive breadth of world models to avoid future mistakes.

Research detected this factor in performance attained on standard tests of academic performance, such as the SAT (Coyle, 2015) or International Educational Competitions, such as PISA, TIMSS, and PIRLS (Rindermann, 2007). There is ample evidence that this factor strongly influences school learning from preschool to university. Psychometric or developmental measures of g account for a large amount of school performance, usually varying around 30% of the variance of school grades or academic achievement tests (Gustafsson and Balke, 1993; Gustafsson, 2008; Kaufman et al., 2012; Roth et al., 2015; Demetriou et al., 2019a,b, 2020a). Notably, g also predicts important life outcomes, e.g., selection of occupation, performance at the job, and income (Gottfredson, 1997; Strenze, 2015), and eminence in many domains, including science and prestigious professions (Bernstein et al., 2019).

#### Learning occurs under the influence of representational and processing enablers

Implementing the processes associated with g above in different domains involves several mediating factors. On the one hand, the state of these factors at any moment constrains representation of information and learning regardless of the domain or symbolic systems involved. On the other hand, these systems constrain how efficiently processes in g may be used. These processes include the following:

1. Representational and processing precision, often expressed in speed of processing (Kail, 2000).
2. Executive efficiency, often expressed as an ability to focus attention on stimuli or representations of relevance to the current goal; inhibit focusing on and processing of irrelevant information, which may pop in because the world is always informationally richer compared to current needs; and relevantly shift between information as the state of problems change by problem solving itself (Brydges et al., 2012; Zelazo, 2015).
3. Working memory, often expressed as a capacity of keeping information active in mind until it is processed according to current meaning making and problem-solving goals.

#### Learning and development gradually transform relational integration processes into reasoning

Reasoning processes develop into Languages of Thought (LoT) expressing the rules underlying distinct forms of relational integration mentioned above. The various forms of reasoning, such as inductive, analogical, and deductive reasoning, enable alignment and abstraction according to the specificities of the information involved and evaluation of accuracy, cohesion, and truth of inferences and interpretations according to rules established until a given point in time.

#### Cognizance

*Cognizance,* i.e., awareness of the objects of cognitive activity at a given moment and awareness that knowledge may emerge from perceptual contact with the world or by inferential inter-relations of perceptions or ensuing representations. This aspect of cognizance is useful for the organism because it frees, partly, processing from the here and now. Being aware of the object of cognitive activity allows to build mental models about it for further use. Also, awareness of cognitive functioning as such and individual cognitive processes enable self-evaluation and self-regulation of cognitive performance (Spanoudis et al., 2015; Makris et al., 2017; Demetriou et al., 2018a, 2019b, 2020b, 2022a).

#### Learning is representation-specific and domain-specific

There is ample research suggesting that *the representational and procedural specificities of different domains knowledge affect learning significantly:* verbal, quantitative, spatial, or social information are differently represented and inter-related (Demetriou et al., 1993; Demetriou and Spanoudis, 2018). These domains are often expressed in preferred symbol systems, appropriate for the relations involved. In language, patterns of sound connected by grammatical and syntactical rules in meaningful sentences are the dominant symbol system (Dehaene, 2010). In mathematics, quantities are expressed in numbers which stand for aggregations of objects or events that may increase, decrease, or re-distribute according to specific rules (Gelman, 1986; Butterworth, 2005, 2010; Dehaene, 2011).

Learning language or arithmetic requires both the general processes above together with domain-specific processes. To parse words in one’s own language and grasp the rules of grammar or syntax, children must decipher recurring patterns of sound, keep them in working memory, match them with memories of them, recognize similarities and differences between word forms (e.g., some-times words end in -ed), and induce relations with actions (e.g., when ending in -ed they refer to actions which took place in the past; Dehaene, 2010). To grasp number, children must discriminate individual objects, recognize their spatial or other relations organizing them into amounts of something (e.g., size and time), keep these patterns in memory and compare them so that their relations standing for numerical operations may be induced (Gelman, 1986; Dehaene, 2011). Even early sensitivity to number, indicating an innate “number sense,” reflects the operation of general mechanisms capturing key characteristics of numerosity in the perceptual field, which generate numerical interpretations (Testolin et al., 2020). However, general mental processes must be able to hold domain-specific representations and relations active for processing to function efficiently (see Demetriou and Spanoudis, 2018; Demetriou et al., 2018b). If, for any reason, children cannot recognize units in a domain, such as words in language or amounts in arithmetic, represent them accurately, and mentally process them, performance in this domain would suffer, even if general mechanisms operate well in other domains.

### Cognitive profiles and priorities change with development

#### Developmental profiles

The general factor, g, is always present, regardless of age or tests used (Carroll et al., 1984; Case et al., 2001; Demetriou et al., 2017). However, *the profile of cognitive processes underlying g changes with development because different processes dominate at successive developmental phases* (Demetriou et al., 2017, 2018b, 2021). The second and third columns in Table 1 summarize the cognitive priorities of successive developmental phases. General intelligence in preschool is primarily based on efficiency in attentional control, awareness of the role of perception for knowledge, linguistic awareness, and awareness of the role of mental states in individual behavior (Demetriou et al., 2022a). Preschool children are attracted by symbols, they explore then systematically, they learn them fast, they use them in their interactions with others, and they understand their dual nature as both *real* entities (e.g., a photograph) and *representations of* something else (i.e., the persons in the photograph; DeLoache, 2000). Play highlights the interest of preschoolers in symbols and symbolic activity as a means of improving the predictive power of their models about the world (Andersen et al., 2022).

Also, language learning and related awareness is important in this phase (Demetriou et al., 2021). Representational insight (“I can think of my friends,” “I can recall what I was doing yesterday”) is a challenge for the toddler: it requires representational control. That is, it requires holding representations active so that perceived information may be encoded and processed according to its relevance to the current goal. *Hence, the priority of this cycle is control of attention so that perception and activity are aligned with represented goals or information.* This is a key developmental task of this phase because it is the background for more complex cognitive tasks, such as setting and sustaining plans of activity, using tools in sake of goals, and exploring objects. Notably, the development of selective attention facilitates development of categorization and concept construction because it allows to search for properties related to a category under formation (Sloutsky, 2010; Deng and Sloutsky, 2015).

Attention control requires awareness. Focusing on one object than on another requires awareness of perceptual systems, such as vision or hearing, which may be directed to objects according to intentions or preferences. Toddlers are aware of their own representations, such as memories of episodes experienced (Paulus et al., 2013). Also, they are aware that perceptions cause representations: people know what they see, touch, and hear. This awareness is the basis of Theory of Mind (ToM; Spanoudis et al., 2015; Kazi et al., 2019; Demetriou et al., 2022a). Children show signs of ToM when they understand that perception generates representations about what they perceive (Demetriou et al., 2018c; Kazi et al., 2019). Also, abstraction and flexibility begin to integrate in this phase: children become able to abstract patterns from distinct stimuli when they can flexibly switch across them and integrate across them according to a common property. Executing a plan of action is possible because it may be deployed based on a common idea running across situations (Kharitonova and Munacata, 2011).

Finally, reasoning emerges in this cycle when event sequences are meta-represented as implicative associations involving a conceived necessary connection between two events. At first, it appears as a meta-representation of “if A then B” relations, laying the ground for modus ponens reasoning. For instance, a 3-year-old seeing dad going upstairs concludes that dad is going to dress, but he cannot explain why. At 5 years, explanation is possible based on awareness of the specific representations involved (e.g., “everyday, dad goes upstairs after breakfast to dress and go to work”). Reasoning in this phase is secondary to attention control and related awareness.

From 7 to 12 years, in primary school, advanced inductive reasoning, simple deductive reasoning, awareness of inferential processes, and working memory define g. With attentional control and representational awareness established, grasping the links between representations becomes a priority. The developmental challenge in this cycle is mastering search of relations between representations and organizing them for efficient recall and later use, in sake of understanding and interaction. Thus, *the developmental priority in this cycle is inferential control* because inference is a major mechanism for grasping relations between representations. Thinking by induction and analogy is the major tool for rule induction and building hierarchies of representations (Gentner and Hoyos, 2017). This allows mental fluency that is evident in analogical reasoning required in arithmetic problem-solving and Raven matrices. Success on these problems suggests that inference becomes fluid in accessing representations (e.g., numbers in arithmetic tasks or figures in Raven matrices), aligning and comparing them, and identifying relations running across them (e.g., each number is the double of the previous one, figures increase). Reasoning is well established in this cycle. For instance, children now understand that the relation “if A then B” necessarily implies that “if not B then not A” (i.e., if an event A causes an effect B, then if the effect is not present the causal event is not present either). Therefore, children grasp relations between reasoning schemes.

Reasoning develops in this cycle together with increasing awareness of inferential processes. Children from 8 to 10 years grasp awareness of underlying inferential processes (e.g., one may conceive of an unknown state of the world by reasoning from similar states); also, they understand that different reasoning tasks activate different cognitive processes (e.g., going around in the city requires visualizing roads; to estimate the cost of goods selected at a shop requires adding up their prize; Kazi et al., 2012, 2019; Spanoudis et al., 2015; Demetriou et al., 2022a). In turn, cognitive awareness motivates inferential awareness and control: inference may be implemented in alternative ways, according to the representations and the problem-solving goals involved, such as planning, induction, or calculation (Kazi et al., 2019; Demetriou et al., 2022a). In short, children in late primary school, organize representations into conceptual hierarchies indexed by language or domain-specific symbol systems, such as visual images or numbers. Rules in this cycle stand out as powerful representations akin to individual representations in the previous cycle. In conclusion, reasoning and related awareness acquire priority in this cycle. Attention control recedes as a predictor it approaches ceiling.

In adolescence, from 11 to 17 years, g is marked by deductive and mathematical reasoning, awareness of logical principles, and increasing precision in cognitive self-evaluation. Thus, *truth, validity, and cohesion control are major priorities in this cycle*. Logic, for those who reach this cycle, becomes important because it is a powerful tool for specifying truth and validity of conceptual and inferential processes (Demetriou and Eflkides, 1989; Demetriou et al., 2017, 2018b; Demetriou and Spanoudis, 2018). This requires specifying general principles underlying relations between rules or concepts. Therefore, control emerges from a metalogical understanding that rules or concepts must be consistent with logical rules. Adolescents do not yield to logical fallacies of deductive reasoning. For instance, adolescents understand that the relation “If there is no petrol in the car the car does not move” does not necessarily imply the relation “The car does not move so there is no petrol” because they can think of reasons other than lack of petrol causing the car not moving. Thus, logical reasoning dominates in this period. Truth control is an epistemic metaprocess specifying when descriptions of reality are acceptable as true knowledge of that reality. For instance, a statement about a relation is acceptable as true (e.g., food A is beneficial for health) only when controlling all confounding variables (e.g., medication, other foods, and exercise); they also have the epistemic understanding that unknown variables may always falsify an assumed relation.

In late adolescence and early adulthood principle-based thought is present across domains. Adolescents solve difficult items in Raven matrices based on a general principle underlying different items. Adolescents conceive of analogies between different concepts based on an underlying general principle. For instance, they can specify multiple relations connecting societal institutions, such as family, education, and government (e.g., father, teacher, and president play equivalent roles in different organizations). Thus, principle-based control enables search across conceptual spaces, their alignment according to assumed relations, and the minimization of differences based on the principles best unifying concepts and relations across the spaces involved.

This hypothetical stance in searching for general principles enables thinkers to associate different representational spaces with principles accepted as true; in this cycle, adolescents understand that truth may vary according to the premises assumed to be true. Thus, consistency in reasoning in this cycle is a product of initial definitions given and the principles selected to connect them. Once selected, they overwrite differences because they shift processing from apparent differences to underlying relations, given the principles. Investing principles to organize other principles may be an infinite recursive process as instantiated in science. Theories in science, such as the search for the theory of everything in physics, or an all-encompassing theory unifying the theory of evolution, heredity, and genetics in biology, are high-level implementations of principle-based thought. Manipulating and communicating principles at this level often needs special languages, such as special mathematics or domain-specific notation. Also, by the end of adolescence, individuals can form long-term life plans, their university studies and career, because they associate choices with preferred principles for organizing their own lives.

The age boundaries of the cycles above coincide with the age boundaries of developmental levels as specified by classic or neo-Piagetian theories of intellectual development (e.g., Piaget, 1970; Bruner, 1973; Case, 1985; Shayer and Adey, 2002). At the surface, this is the case. However, these cycles index “regions of change in the profile of interacting processes” rather than hard boundaries of distinctly different abilities. These are time windows in which different developmental priorities dominate, according to the processes that must be mastered, if dealing with the world would become increasingly successful. However, performance may not necessarily be uniform across domains. Developmental priorities satisfy different adaptive needs, such as sustaining interaction with objects in infancy, sustaining attention to regulate one’s own action according to goals in early childhood, integrating and filling in gaps in information and knowledge in the age of primary school, and checking for truth and cohesion in adolescence and early adulthood.

### How change happens

#### A factor of change on top of g

On the one hand, g in each cycle gears on a different combination of the processes above, according to each cycle’s priorities. On the other hand, implementing developmental priorities creates dynamics of change on its own which is distinct from the current state of each of the processes involved. Notably, cognizance is a stable component in the process of satisfying developmental priorities. In each cycle, cognizance reflects the processes dominating in the cycle, suggesting that cognizance is part of the transition force underlying progression across cycles.

A recent study differentiated the ability at first testing from the momentum of change from first to second testing, 2 years later. To implement this differentiation, a second-order g factor was associated with all domain-specific factors representing attention control, working memory, reasoning, and cognizance. To capture change as such, a second general factor was associated with the difference between performance at first and second testing. This factor was used to predict change from first to second testing in each process, in addition to g underlying performance at first testing. The change factor predicted change in executive and reasoning processes more than g at first testing (Kazi et al., 2019). Also, this factor indicated that distance from the final level of a cycle was positively related to the size of change: the larger the distance from the final state the larger the magnitude of change across processes, reflecting a general developmental momentum towards the consolidation of a cycle and transition to the next cycle. Cognizance of the processes dominating in each developmental cycle was the major predictor of this momentum. Interestingly, there is evidence that a general factor for cognitive change operates in late adulthood as well (Tucker-Drob et al., 2019).

Striving to meet the developmental priorities of a cycle causes learning related to these priorities. At preschool, trying to attain attention control results into learning the role of perceptual processes. For instance, focusing on a stimulus of interest and avoiding looking at another attractive stimulus makes the child realize that information comes from the senses and that she may control, if focusing on them; for instance, she may persist in looking at and thinking about a goal-related stimulus while effortfully ignoring others. In turn this may make the child realize that differences between persons in perceptual access to a specific stimulus cause them to have different knowledge about it. Later, striving to attain inferential control by combining information may make children realize that inference fills in gaps in information.

### Training general cognitive processes.

Research examined if training relational thinking, deductive reasoning, and awareness of the processes involved may increase fluid intelligence and transfer to other processes, such as attention control and working memory. One study examined if training relational thought and awareness of comparative and integration processes would improve mathematical problem solving in district domains of mathematics and generalize to other mental processes (Papageorgiou et al., 2016). This study involved 10-year-old children who were at fifth grade in primary school at first testing. Children had to identify relations underlying number series (e.g., double, triple, half, and one fourth) and mathematical analogies, specify their similarities and differences, organize them into hierarchies of rules, and solve problems involving all relations. Thus, they learned to explicitly represent problem structures and processes at successive levels of abstraction and identify their similarities and differences.

Children were examined on general cognitive processes (i.e., attention control, working memory) and different forms of reasoning (i.e., deductive, analogical, causal, spatial, and algebraic reasoning), in addition to mathematical problem solving. Mathematical reasoning, the target of training, significantly improved at the end of the intervention and improvement lasted until 6 months later. Also, the gains transferred to domain-free analogical reasoning, highly related to the training of relational thought, but also to other less related domains of reasoning, such as deductive reasoning. This improvement was weaker but stable in time, implying transfer from mathematics to general reasoning processes. Notably, working memory and attention control also improved across testing times. Therefore, training core relational processes and their explicit awareness transferred the gains of learning to general executive and reasoning processes.

A second study examined how awareness of the logical relations in deductive reasoning schemes and skill in building mental models for them would cause transition from rule- to principle-based reasoning (Christoforides et al., 2016). This study trained third (8 years of age) and fifth (11 years of age) grade primary school children to become aware of the logical relations underlying the four schemes of conditional reasoning (i.e., modus ponens, modus tollens, affirming the consequent, and denying the antecedent). Children were also trained to construct mental models appropriate to represent the logical relation underlying each scheme. Thus, this study examined if enhancing cognizance of inferential processes involved in each scheme would enable children to acquire the principles underlying all four schemes. The study also explored if progress from scheme-limited rule-based reasoning to the general principles of deductive reasoning relates with attention control and working memory. There were three groups: (1) Control, with no training. (2) Limited instruction, aiming to enable children to grasp the notion of logical contradiction and the logical structure of each scheme. (3) Full instruction, aiming to enable children to adopt an analytical approach to logical arguments; these children were trained to differentiate between the stated and the implied meaning of propositions and take the meaning of propositions as given for the sake of the argument, regardless of the “every-day” use of the terms; e.g., if we accept that “dogs fly” and that “Rex is a dog” we have to accept that “Rex flies” regardless of what we know about dogs and Rex. Also, training in this group aimed to enable children to identify truth and logical contradiction and understand the notions of logical necessity and sufficiency.

This study enabled children, in about 3 weeks, to traverse a full developmental phase, normally lasting for 3 years. Third graders in the full instruction group solved problems requiring principle-based reasoning *if supported by context*; sixth graders in the full instruction group attained this level regardless of content and context. That is, both age groups mastered the fallacies of affirming the consequent and denying the antecedent. Awareness of the inferential identity of each scheme and logical consistency were critical for this success. Notably, explicit awareness improved significantly only in the full instruction group; grasp of this awareness related with attention control and working memory. There was an interesting difference between third and fifth graders: third graders implemented the logical principles, but they did not explicitly state their formal characteristics; sixth graders both specified the principles explicitly and performed accordingly.

### Learning is developmentally specific

Psychometric g is a major predictor of school performance (Gustafsson, 2008; Ritchie and Tucker-Drob, 2018). However, the present theory predicts that the contribution of different processes in g to school success varies as a function of developmental priorities: *the best cognitive predictors of academic achievement at different developmental periods are the mental processes prioritized in each period.* A series of studies evaluated this prediction, examining children from 5 to 18 years of age, i.e., from preschool to late secondary school.

In line with this prediction, these studies showed that attention control and perceptual awareness are the major predictors of school success in preschool, from 4 to 6 years. The state of these processes at preschool also predicts school success 4 years later, in primary school. Notably, the momentum of change across testing waves from 4 to 6 years was a strong predictor of school achievement years later, at the end of primary school (Demetriou et al., 2020a). A second study found that working memory, cognitive flexibility, and inductive reasoning were the best predictors of school performance in primary school (Demetriou et al., 2020b). In line with these findings, research showed that working memory and fluid reasoning accounted for a large amount of variance in mathematics and language in late primary school (*circa* 30%) but the influence of self-esteem and mindset was much lower (often *circa* 1%, if at all; Giofrè et al., 2017; Vernucci et al., 2021). Expectedly, interference control and working memory ceased to predict school performance from seventh to ninth grade (Dubuk et al., 2020), implying that the time window of their predictive strength closed. In adolescence, competence in language, deductive reasoning, and accuracy in cognitive self-evaluation were the best predictors (Demetriou et al., 2019a). Also, conscientiousness, one of the Big Five Factors of personality, was a significant predictor of school grades in this period. This factor indicates if one is goal-minded, focused, organized, determined, and self-controlled (MacCrae and Costa, 1999). Conscientiousness is a refined expression of executive control in adolescence and adulthood (Demetriou et al., 2022b).

Studies examined if changing specific aspects of general cognitive processes would transfer to school performance, if aligned with the developmental priorities of the phase involved. Espy et al. (2002) showed that training inhibitory control was central in accounting for learning mathematics in preschool. With entrance to first grade, working memory became central. Monette et al. (2011) showed that working memory and inhibition in kindergarten predicted learning to read and write and arithmetic at the end of first grade. Later, executive mechanisms enabling focusing and manipulation of information when learning emerged as central. A longitudinal study involving first- and second-grade children showed that updating in working memory but not inhibition and shifting predicted learning mathematics. Moreover, changes in memory updating and mathematics learning were related: working memory and updating facilitated mathematics learning and this facilitated both executive functions (Van der Ven et al., 2012). In conclusion, these studies showed that mastering executive processes and representational awareness in preschool is critical for school learning in both preschool and primary school. When prediction draws on process mastered in primary school, working memory and inductive reasoning dominate as predictors. Later, in adolescence, more abstract aspects of reasoning, self-awareness, self-evaluation, and self-regulation dominated as predictors (Demetriou et al., 2022b).

There are good educational reasons for this pattern of relations. Executive processes are important for learning in preschool and early primary school because they enable children to focus their learning competence on school-important skills and concepts, such as reading and arithmetic (Chung and Ho, 2010; Clark et al., 2010; Nelson et al., 2017; Vanbinst et al., 2020). In primary school, understanding the role of inference in knowledge and learning facilitates school success because it allows self-directed use of cognitive processes for learning the complex concepts and skill taught in primary school. In adolescence, emergence of principled-based reasoning and accurate self-awareness and self-evaluation aligns with the semantic, syntactic, and abstract reasoning demands of the scientific concepts and problem-solving taught at secondary school (Demetriou et al., 2018a, 2020a).

## General problem solving

There is a problem to be solved when a ready-made solution is not available. Broadly speaking, problem solving is figuring out how to attain a goal for which no ready-made solution is available. Technically speaking, problem solving implies efficient use of g which would implement the possibilities associated with a given state of g. Overall, problem-solving involves the following discrete components:

1. Using cognitive competence and knowledge to understand what a problem is about.
2. Specifying where in a problem available knowledge is not enough for the goal.
3. Exploring if variations of existent knowledge or strategies may meet the goal.
4. Search for other knowledge, information, or strategies that may serve the purpose.
5. Invent a solution by inferentially extrapolating from present knowledge, creatively and validly filling in gaps in old knowledge.
6. Evaluate solution for adequacy and accuracy and properly encode this solution for future use.

Cognitive developmental priorities of successive developmental cycles constrain the situations presenting problems that may be solved and the solutions possible (Stadler et al., 2015). The respective column in Table 1 summarizes changes in problem-solving possibilities with development. Mastering interaction with objects is a major source of problems in infancy. The lack of differentiated representations that infants might use to specify a goal and plan a sequence of steps for solution limit the infant to trial-and-error strategies which allow a gradual modification of an initial behavior, such as using a spoon to eat (Keen, 2011).

In preschool, mastering executive control is a major source of problem-solving opportunities. Preschoolers need to realize that alternative representations may stand for the same thing and for each other. To solve problems, they need to learn how to build a representation for a problem (e.g., jumping from the sofa on the floor), plan their actions (e.g., contraction of feet and hand in a specific way before jumping), and evaluate (e.g., if it was precise, if it was painful, etc.) in order to improve next time (Zelazo et al., 1997). Thus, mastering strategies to resist temptation or distraction, stay focused on goal, and produce a solution is important in preschool. Early programs training attention control and related perceptual awareness and associating them with school-related activities would help learning reading and arithmetic because these learning domains require extensive attention control and awareness at their initial stages.

In primary school, children need to learn that they can “decipher” missing representations from other representations, when their relations are known. Thus, they must learn how to recruit available knowledge, look for new knowledge if available knowledge does not suffice, and use reasoning to relate representations based on object properties (as in arithmetic problem solving and text comprehension).

Later, adolescents must realize that they may generate sets of representations for a given object provided the constraints given. Adolescents must understand that problem solving is a dynamic interaction with task situations which change as a function of the own interventions aiming to reveal their regularities emerging from ongoing explorations and their integration into rules and solutions (Frensch and Funke, 1995). Expectedly, problem solving is moderately related with general intelligence (Stadler et al., 2015) and *critical thinking.* Critical thinking requires viewing representations, concepts, believes, and action models from the point of view of each other, evaluating their value for the individual and the group, and specify alternative conditions under which they might be acceptable. Thus, critical thinking is a general strategy for maximizing use of general intelligence *because* it draws on cognitive competence available to generate alternative options and evaluate them against each other, drawing on knowledge available or information currently flowing in the environment (Sa et al., 1999). From the point of view of the present theory, creativity is an exhaustive application of critical thinking to create and explore variations of solutions which, on the one hand, seem promising to solve a problem and, on the other hand, solutions are novel enough to alter the possibilities of facing the problem again in the future.

## Learning in language and mathematics

We showed that transformations in cognitive ability relate with changes in the relative state and interactions between different cognitive and representational processes, including facility in using different forms of representation and enabling processes. Here we focus on two important domains of school learning: language and mathematics. The respective columns in Table 1 summarize changes in developmental priorities for learning in these two domains. Teaching in both domains starts at preschool and continues through college. There are similarities and differences between these two domains. On the one hand, they are both highly symbolic, requiring mastering arbitrary codes, specified at several levels, and related by specific rules. On the other, the two systems differ in what they represent and how, such as grammatical and syntactic rules in language and magnitudes and numerical operations in mathematics. Language is a universal system which children learn since birth (Dehaene, 2010). Mathematics appears later when schooling begins, even if mathematical concepts appear in infancy. Notational and representational systems in mathematics differ extensively from the systems in language. Also, the rules governing transformation of representations in the two systems differ extensively: grammar, syntax, and semantics in language as contrasted to mathematical notation, arithmetic operations, and rules in mathematics. Children learn to speak in infancy and to write in middle childhood. In mathematics children recognize numbers and simple numerical transformations since early in age but learning formal mathematics is a prolonged process developing through secondary school.

Thus, it is important to specify similarities and differences of learning in these two domains. This theory suggests that efficient learning in each domain depends on tuning domain demands for learning with developmental priorities. Learning difficulties emerge any time school demands for learning in a domain diverge from children’s possibilities. Delays in mastering developmental priorities in preschool (attention control and representational awareness) would cause difficulties in learning the symbolic skills required by reading or arithmetic in early primary school. Deficient representational awareness would impede children in grasping the representational nature of written words (i.e., written words stand for oral language). Deficient attention control would impede children in focusing on, registering, and encoding letters or numbers. Delays in mastering rule-based thought in primary school may cause difficulties in reading comprehension based on grammatical and syntactical rules or in implementing arithmetic operations on numbers. Delays in grasping principles underlying relations between rules would cause difficulties in grasping abstract science concepts, such as energy, gravity, or evolution. Therefore, educators must diagnose deficiencies in time to be able to remove them before they cause learning problems in other domains. The proposals below provide a general framework for the implementation of the aims of school curriculum by coordinating priorities in the selection of concepts and skills in language and mathematics with cognitive developmental priorities; this would increase the efficiency of teaching more than is presently possible.

### Language

Reading involves three hierarchical levels (Kintsch, 1988, 1994; Kintsch and Rawson, 2007) that align with the developmental cycles described above. The first level requires encoding dominated by perceptual attentional processes. All alphabetic systems of writing require to learn how letters stand for sounds, how they are composed to produce syllables which, in turn, compose words arranged in sentences, according to grammatical and syntactic rules. Learning at this level starts in preschool, when children recognize script or words as symbols for speech and they engage in pre-writing activities aiming to master the skills required for writing. Learning to read and write is a major goal at first and second grade of primary school.

According to this theory, learning to recognize letters and compose them into syllables and words is based on the developmental priorities of the cycle of realistic representations: (i) Attention control is important for learning to read, because the integration of letters into words and words into sentences requires focusing and shifting attention. Evidence shows that deficiencies in control of attention allowing systematic spatial search at the initial stages of reading hinder learning on top of limitations in IQ, hyperactivity, and other behavioral problems (Rabiner et al., 2000; Franceschini et al., 2012). These difficulties are present in different languages, such as Arabic (Friedmann and Haddad-Hanna, 2014) and Chinese (Chung and Ho, 2010). (ii) Representational awareness is important to recognize words as signifiers of objects or actions one may observe, process, and inter-relate mentally. Representational awareness, expressed here as phonological awareness and comprehension monitoring, allows children to monitor, reflect on, and evaluate their comprehension to reprocess and re-visit the text whenever necessary. There are mutual causal relations between phonological awareness and reading achievement: phonological awareness enhances literacy development which results into further growth of phonological awareness (McCardle et al., 2001; Farrar et al., 2005). Representational awareness mediates between working memory and listening comprehension (Kim, 2015). In conclusion, representational awareness is a top-down mechanism guiding visual search and integration of representations into blocks of meaning.

The second level goes beyond recognition and production of words to abstraction of meaning from them. At this level, readers construct a textbase representation, drawing on language and cognitive processing mechanisms. Textbase representations require vocabulary and syntactic knowledge that would enable children to abstract meaning from words as they are combined into sentences. The actual words in a text may not be enough for the construction of an accurate and coherent textbase representation. Thus, the third level goes beyond words, importing assumptions about relations between sentences which are used as mental models of the situations imposing top-down meaning on words and sentences (Kintsch and Rawson, 2007, p. 211). At this level readers form causal inferences about the actions and relations in the story, drawing on past knowledge or reasoning to fill in gaps in information or grasp nuances not directly spelled out by the words involved.

Reading at the second level starts at second primary school grade and it may continue until fourth grade for many children. In terms of the present model, this level draws primarily on rule-based thought that allows to discover threads of meaning running through strings of sentences. The third level is based on both, rule-based and principle-based thought. General principles and prior knowledge may be invoked to infuse meaning on top of the literal meaning of the words involved. As expected, working memory and fluid intelligence predict reading comprehension at the second level by third graders (García-Madruga et al., 2014). Comprehension in adolescence draws on that advanced principle-based thought (Makris et al., 2017; Demetriou et al., 2019b).

International assessments of literacy, such as PISA (2012), address these abilities. These assessments assume that children are able, by second primary grade, to link multiple pieces of information to draw inferences about events and relations and integrate information across a whole text to identify a main theme and related ideas. By sixth grade, children must be able to grasp the central idea in a text, even when multiple events or themes are described, shadowing the central idea. Children must be able to decipher a hierarchy of ideas and specify their connections. By third secondary school grade adolescents must be able to abstract common ideas running in different texts, such as newspapers, scientific texts, and literature. Only about 15% of third graders in OECD countries perform at this level.

Therefore, school curriculum must implement the following principles for language which are based on the model of developmental priority proposed here:

1. Facilitate children to understand that the flow of sound in their everyday language may be expressed in a flow of visual symbols which they can read or write themselves.
2. Enable preschoolers and early primary school children to realize that details matter because written language has constraints corresponding to the constraints of oral language. Thus, attention in visual or writing activities is important for learning to read and write.
3. Enable primary school children to explore different language units, their composition into elements, and their relations to understand that specifying discrete elements in different language units is important for abstracting meaning from texts.
4. In late primary school, children must grasp syntactic and grammatical rules as independent from specific texts, their content, and the context in which they are used.
5. In late primary school and early secondary school students must explore relations between diverse ways of representing the same reality, such as pictures, movies, alternative verbal descriptions and specify the differences in the meaning they convey. The role of inferential processes must become apparent in these explorations.
6. In late primary secondary school, education must enable students to grasp the principles underlying differences in style and form between various aspects of language use, such as personal communication, media communication, literature, and science.

### Mathematics

Internationally, the mathematical curriculum includes five major domains: number-operations, geometry, measurement, algebra, and statistics-probabilities. The National Council for the Teaching of Mathematics (National Council of the Teachers of Mathematics, 2000) specifies mathematical targets for each school grade that students must master across these five domains in five major realms of cognitive activity: reasoning and proof, problem solving, representations, communication, and connections. Students should be able to mentally create representations and use them to express, organize, and communicate mathematical ideas, transform them across content domains, and conceive of the physical, social, and mathematical phenomena implied by mathematical expressions. In this paper we focus on numbers and operations which underlie all domains of mathematical thinking.

The Approximate Number System is a major mental background for the development of mathematical reasoning and learning school mathematics (ANS; Dehaene, 2011). Subitization is a foundational core process in ANS: this is the automatic perception of the number of small sets including no more than 3–4 objects. Subitization appears early in infancy, and it is present in other animals, such as birds and mammals (Dehaene, 2011). Infants also recognize basic arithmetic operations, such as addition or subtraction, when performed on small sets. In combination, these processes develop into the mental number line in early preschool. The mental number line is a general mental template of the relations between numbers, arranged from left to right according to magnitude. This template is mentally accessible at will, allowing fast (and often approximate) comparisons between numbers. Comparisons are easier and more accurate with small numbers or small distance between numbers. For instance, it is easier to specify that 17 is larger than 13 than to specify if 877 is larger than 865.

The APN and the mental number line develop throughout childhood and adolescence (Dehaene, 2010; van Marle et al., 2013; Siegler and Braithwaite, 2017). The mental number line appears between 3 and 5 years, covering only small numbers between 1 and 10. Preschool children can map number words on small sets of up to three dots, but not on arrays of 4–6 dots; also, they cannot compute arithmetic operations (Benoit et al., 2013). They have a global representation of quantities in small sets, and they can associate them with corresponding number words. Later, from 5 to 7 years, the mental number line extends to 100 elements. In this phase, children can align representations from different representational spaces, such as toy cars and toy children, and they can match number words on number digits in arrays of up to six elements. Also, at 5 years, children can compute additions and abstractions on numbers smaller than 10, using their fingers as tools for counting. Counting includes both reciting a series of numbers and understanding a symbol cognitive parameters of the development of mathematical thinking in early childhood.

Attention control and phonological awareness are strong predictors of learning in arithmetic. In preschool, individual differences in control of attention, goal-based shifting between representations, and planning relate with individual differences in learning arithmetic operations (Clark et al., 2010). Also, phonological awareness predicts learning of early reading and early arithmetic; inversely, number recognition predicts performance in both domains (Vanbinst et al., 2020). Obviously, general representational awareness is the key factor rather than awareness about a specific notational system.

The school curriculum in mathematics must implement the following principles which are based on the model of developmental priority:

1. Facilitate children to explore magnitudes, their composition into elements, and their relations to understand that specifying discrete elements in different magnitudes (sets) is important for further processing.
2. Grasp basic properties of numbers, such as that they are independent of the physical properties of the elements involved.
3. Explore relations at different regions of the mental number line to realize that relations between individual numbers are independent of their size.
4. Explore relations between diverse ways of representing numbers, such as number names, number digits, and other representations, such as figural and geometrical notation.
5. Gradually induce students into the value of using notation for mentally manipulating numbers and their relations.

Therefore, the curriculum in mathematics must establish number awareness and ensuing number sense in preschool and early primary school: a general understanding of numbers, operations on them, and the use of strategies for solving problems involving numbers (Way, 2011). Individuals with a good number-sense grasp numbers intuitively, count, grasp quantitative relations, plan operations on them, shift between them, and associate numbers and operations with symbolic representations (Mohini and Jacinta, 2010; Ontario Ministry of Education, 2016). Children younger than three may have number sense, albeit global: they may count using number names; if asked what number comes after 4, they may start from 1 and continue until they state the number coming after 4. Also, they may count objects without systematically associating each number name with pointing to the next not counted object; thus, they may end up with different numbers when counting the same set, indicating a fragile concept of cardinality: that an amount is always the same if specified in reference to the same set of objects and the last number stated when counting indicates the number of objects in the set counted.

Hence, school curriculum for early preschool, up to 4 years of age, must enable children to precisely connect the value of numbers with counting acts. A first grasp of the relations between numbers is mastering common global quantitative comparisons, such as “more,” “less” and “same as.” Understanding quantity comes next, when understanding cardinality. At 4–6 years emphasis must shift to aligning and connecting complementary representations of number, such as number names (e.g., 1, 2, 3, … 10), corresponding symbols (e.g., 1, 2, 3, …, 10), and the quantities represented (e.g., one thing, two things, three things, …, ten things).

The number line extends to 100 between 5 and 7 years. In this phase, children can align magnitudes and numerical symbols from different representational spaces, such as number words and number digits, but alignment is not fluid yet. Therefore, in this phase, children need experiences in using a variety of tools and resources (e.g., number lines standing for different categories of objects) to realize that the number system is pattern-based where numbers may recur, always having the same value (e.g., the sum of 3 + 5 = 8 is always the same regardless of how many tens are involved: e.g., 23 + **5** = 28, and 33 **+** 5 = 38). The rules underlying these relations must be explicitly represented, matching the rule-based character of thought in this phase. Also, children need to use manipulatives and pictures to represent mathematical concepts before the introduction of symbols, thereby building the necessary links between the mathematical concepts and the symbolic language which may stand for them. The introduction of symbols as a mean of communication and representation is important for preschool education because it allows the consolidation of representational thought. A quantity of seven objects can be represented by seven objects, seven pictures, seven images, or seven points on a line. Also, they can compute additions and subtractions on numbers smaller than 10, using their fingers as tools for counting or other manipulatives or images. The use of different representations for the same concept facilitates representational awareness and abstraction underlying rule-induction.

At the first primary school grade, emphasis must shift to the exploration of the patterns between one-digit numbers, tens, and decades, to grasp the rules underlying the structure of place value system (Kilpatrick et al., 2001). For instance, TEN is an important anchor at this age because it helps children to remember the combinations that make 10 (3 + 7) and that two-digit numbers, e.g., 12, is divided into 10 and 2, and 25 may be decomposed into 2 × 10 and 5 units. Number sense is enriched when grasping place value. A numeral represents a number symbol, a word, a position on the number line, a quantity, and a place value position (e.g., 2 can mean 2, 20, and 200 in respect to the place value). Thus, first grade children must grasp that a general rule connects various aspects of number; also, that knowing this rule allows transforming numbers to each other, according to this rule. Grasping this rule requires that children in Grade 1 and 2 need opportunities to experience counting to 100 and to establish links between the numbers and their visual representations as numerals. They must be able to use their understanding of relations along the number line to calculate objects in sets and place them in correspondence. Second grade children must be able to estimate what they can buy among a set of desirable objects, given the money they have. Also, they must be able to translate simple word problems into mathematical expressions.

From 7 to 12 years the mental number line extends massively, going up to 1,000 at fourth Grade and 10,000 at fifth Grade. This expansion is associated with understanding the rules underlying the relations between numbers and their transformation into each other. Thus, in Grade 2, children must understand that if 6 × 8 = 48 then 7 × 8 is 48 + 8. In Grade 3, numeric relations and patterns must be used to develop relevant strategies. For instance, they can think of “68–29” by relating it with “69–30” and make the calculation easier. Children must understand subtraction as the inverse of addition and division as the inverse of multiplication. Also, they must understand that division may be translated into repeated subtraction and multiplication may be translated into repeated addition.

In the following grades, counting may extend to large numbers. Normally, at this phase, children must be able to extrapolate their early grasp of the relations between numbers to large numbers (e.g., the relationship between 2 and 5 is like the relationship between 22 and 25 and between 1,232 and 1,835). Obviously, early number sense becomes highly refined at the final grades of primary education. However, children must have practice to develop strategies for mental calculations that would generate exact numerical results and relations beyond the familiar small numbers (Yang et al., 2007). At this phase, the recognition of relations between numbers must extent from integers to rational numbers and complex numbers.

Fluid mathematical thought requires that consolidation of number sense with integers must expand to include rational numbers. For instance, the ability to accurately place numbers on a number line at third grade is a strong predictor of understanding fractions at fourth grade, indicating that grasping general relations between numbers allows mastering their most complex expressions and the specificities of their transformation in each category (Jordan et al., 2016). Fraction involves an understanding that whole numbers are divided into equal parts according to the numerator and the denominator. Thus, the shift from whole numbers to rational numbers must expand from the activities with discrete quantities to activities with continuous quantities. Understanding fractions is a demanding process, because children face significant difficulty to think of fractions in terms of symbols before the end of primary school (from fourth to sixth grade). However, using fractions in primary school is needed for success in more advanced mathematics, such as using algebra in secondary school (Booth and Newton, 2012; Siegler et al., 2013).

Grasping the relations between operations on whole numbers and operations on fractions requires understanding that algebraic numbers are variables that can take any value and that operations on them depends on their precise nature (e.g., whole numbers vs. fractions or decimals). This is attained in adolescence when the mental number line is conceived as a sequence of any numbers to infinity. In secondary school, adolescents can solve problems which require this general conception of number and the assumption of constraints that define the value of number instantiations, given their relations as specified in mathematical propositions. For instance, they can understand that “a + b + c = a + p + c,” if a = p (Demetriou et al., 1996; Demetriou and Kyriakides, 2006). As a result, numbers may be defined in alternative ways (e.g., natural, real, and imaginary number); their relations can be explored if they are consistently defined according to general principles underlying relations between rule-systems operating in each category (Dehaene, 2011). By third secondary school grade, adolescents must be able to represent complex situations in formal mathematical language, and specify their similarities and differences given the formal properties of the representation selected, such as natural numbers, fractions, or decimals (Ni and Zhou, 2005; Shiel et al., 2014, 2016).

### Problem solving, mathematics, language, and metacognition

Problem solving is central in learning mathematics. The aim is to introduce learning in number, algebra, and geometry through real-life scenarios and through scenarios related to different scientific fields, such as physics and biology. These activate cognitive processes to make meaning of unknown situations, express meaning and represent distinct aspects of the world or domains of knowledge in the language and problem-solving of mathematics. Selection of relevant previous knowledge and strategies to represent a problem mathematically depends on multiple factors such as the relations involved in the real world or distinct fields of knowledge, their complexity, the cognitive load they impose, and obviously the mathematical operations needed to specify the unknowns of the problem. For instance, solving word problems requires language comprehension to build a first representation of a given reality and the conventions or symbol systems of different forms of relations. Students must use vocabulary and text comprehension skills to derive meaning from the text and understand the academic and mathematical vocabulary (Fuchs et al., 2014).

Also, students must draw on inductive and deductive reasoning to systematically fill in missing information and evaluate interpretations based on the givens of the problem and earlier similar problems and solutions given, and computational facility in implementing specific numerical operations as required. Often, students must relate data retrieved from alternative types of representations and present their solution in an acceptable mathematical form. A recent meta-analysis of studies of word problem solving showed that language, working memory, attention, mathematics vocabulary and mathematics computation emerged as unique predictors of successful performance (Lin et al., 2020; Verschaffel et al., 2020).

Students need to select the data, understand the context of the problem, select an algorithm or a procedure to obtain an appropriate solution, and apply cognitive strategies to evaluate the logical relevance of their answers. At each stage of the problem-solving process, students need to self-regulate their performance, evaluate their actions in respect to the goal, justify and explain. These processes require awareness which varies at successive developmental phases. People extend their problem-solving skills by exploring their limits and reflecting on them. This requires training students of different grades to learn a new mathematical procedure and then explore the solution of unfamiliar problems using the same procedure or learn a different procedure for the solution of the same problems (Anderson and Fincham, 2014).

### Dyslexia and dyscalculia: Understanding developmental learning difficulties

A recent meta-analysis of many studies showed that fluid intelligence was moderately related to both reading and mathematics (*r* = ~0.40); also, learning to read and do arithmetic boosts fluid intelligence (Peng et al., 2019). This relation explains why difficulties in each domain relate with difficulties in the other domain, indicating dependence on common representational-processing mechanisms. Thus, at transition from preschool to kindergarten, from 4.5 to 6.5 years, executive functions interact with each other and with early numeracy and literacy skills (Schmitt et al., 2017) and listening comprehension predicts numeracy skills (Aunio et al., 2019). The last column in Table 1 summarizes learning difficulties in language and mathematics across developmental phases.

Despite their commonalities, the two domains have special characteristics as well, which cause specific learning difficulties in each. In language, about 20% of children in early primary school have difficulties in learning to read and write; *circa* 5–10% of these children satisfy the requirements of dyslexia. Dyslexia is a serious condition because it interferes with every aspect of school life blocking learning dependent on reading. Dyslexic children fail in phonological processing, which is important for the translation of letters into sounds and their integration into words (Catts et al., 2001; Siegel, 2006). Notably, about 6% of children also face developmental dyscalculia (Reigosa-Crespo et al., 2012). These children “have a poor intuitive sense of quantity, … poor understanding of more and less, and slow learning of Arabic numerals, number words, and their meanings” (Chu et al., p. 9). They cannot fluently enumerate objects in small sets of up to nine elements, they cannot compare small amounts, such as 5–7, and add or subtract one-digit numbers. Butterworth (2005) proposed the “defective number module hypothesis,” suggesting that dyscalculia is caused by deficient numerosity coding. Numerosity coding is mapping symbols onto representations of quantities, allowing an exact representation of number symbols or names in reference to their corresponding quantity (e.g., 1 as a quantity of one, 2 as a quantity of two, etc.) A deficit in numerosity coding would make learning to count difficult because counting words would lack the exact corresponding representations to be associated with, thereby blocking the formation of the ANS, and grasping the relations between quantities. Numbers are mixed up on the number line because they often overlap in dyscalculic children (Mussolin et al., 2010).

Reading difficulties, including dyslexia, and mathematics difficulties, including dyscalculia, appear to share a common representational deficit. They both depend on a background of general attentional difficulties. However, they often have representational difficulties specific to each representational system. In reading difficulties, the phonological system lags the resolution required for letter identification. In arithmetic difficulties, numerocity coding lags the precision required to build representations of different quantities. Dyscalculic children cannot easily associate Arabic numerals with corresponding magnitude representations; however, they have no problems in associating letters with phonemes. Dyslexics have problems with letter and digit recognition and naming; however, they do not have problems with magnitude processing (Rubinsten and Henik, 2009). Notably, word inhibition in the Stroop task (i.e., recognize the ink color of color words, such as RED, written in a different ink color, such as blue) suffers in dyslexics and number inhibition (e.g., recognize which number is bigger, 8 or 5) suffers in dyscalculics. Children with both dyslexia and dyscalculia face cumulatively the problems of both groups (Landerl et al., 2009). Interestingly, graph inhibition, which is the recognition of a geometric shape in the context of more complex geometric figures suffers in both groups, implying a more general attention control problem that is aggravated in domain-specific symbol systems in each group (Wang et al., 2012).

Difficulties in learning written language and mathematics depend on both, encoding and representation of domain-specific information and general representational and processing functions. All domains need both domain-specific and general representational ability to operate above a certain level for learning to occur smoothly. Severe domain-specific representational difficulties may seriously interfere with learning in the domain concerned, such as phonological and magnitude representation in the domains of language and arithmetic, even if general representational and processing functions are intact. However, smooth, and efficient learning in all domains requires both, domain-specific and general representational functions to operate above a certain limit.

## Conclusion

We outlined a theory unifying cognitive, developmental, psychometric, and clinical research on intellectual development and learning. The basic postulates of this theory are as follows:

1. Cognitive development occurs in cycles of representational change; each cycle prioritizes distinct types of representations and different relations between them, allowing an increasingly accurate multidimensional representation of the world. Progression across the cycles transforms the very nature of general intelligence, g. In the present context, g is considered as a machine generating world models. These world models become increasingly complex and predictive across cycles, allowing learning of increasingly complex skills.
2. New forms of representation and world models in successive developmental cycles demand new forms of mental control, enabling their efficient handling. Attention control in preschool allows focusing on mental representations and associates them with actions. Control of inferential processes in childhood allows capturing relations between representations and using rules for the expression of these relations. Logical control in adolescence allows to evaluate truth and cohesion of representations and the rules underlying their relations.
3. Mental awareness is a key component of these changes. In each cycle, it reflects the representations dominating: representations and their perceptual origins in preschool; inference and strategies allowing to organize representations, such as categorical organization and rehearsal in working memory in late primary school, and logical principles and their symbols, such as connectives in adolescence. In evolutionary terms, awareness may have evolved to compensate for the limited capacity of the brain to build models about the world. It helps focus on the most valuable information relevant to a current problem to build a model for it, dropping irrelevant information. Thus, changes in awareness reflect changes in the processes that must come under control.
4. Control is exercised according to the symbol systems used; symbol systems stand for distinct levels of mental complexity and may express the same aspects of reality at distinct levels of resolution. Control of attention is first needed to enable focusing on relevant information. Becoming aware is also important when representations dominate as mediators between individual action and the world because it enables choosing among models, one’s own or others,’ and negotiating with others. In the next cycle, prioritizing storage of large amounts of information is important. Thus, at this phase, working memory and inference take priority for efficient building of world models. Finally, a systematic validation and cost reduction is prioritized in adolescence to optimize selection of the best possible world model.
5. Commanding a learning domain requires facility with the symbol systems involved; for instance, acoustic patterns standing for spoken words and visual patterns standing for written words or quantities. If grasping and representing patterns in the environment is deficient, their learning would also be deficient, as in speech delays at the transition from infancy to early preschool or reading and arithmetic difficulties at the transition from preschool to secondary school.

Individual differences in mastering major developmental tasks above occur at transitions across developmental cycles. All three major transitions are associated with fast learning of a new symbol system. Speech at the transition between episodic representations in infancy and realistic representations in preschool. Reading and writing at the transition from realistic representations in preschool to rule-based thought in primary school. Highly specific idiosyncratic symbol systems at the transition between rule-based thought in primary school to and principle-based thought in secondary school. From an evolutionary point of view, the three systems at separate times in history. Human language emerged with homo sapiens at about 200,000 years BC; reading and writing is about 5,200 years old; domain-specific symbol systems, such as modern mathematical notation, is a recent attainment. Mastering each system becomes increasingly difficult and learning difficulties increasingly likely. Most children speak at 2–3 years of age without education. Most children learn to read and write at 6–7 years, but only if specifically educated. Mastering mathematics or other sciences demands long education (Demetriou, 2020b).

In all cases, difficulties arise when central and domain-specific processes do not develop at the same pace. Speech delays may occur either because children do not have the capacity to manage language complexity during encoding (Panagos et al., 1979) or because of specific phonological encoding problems (Paul and Shriberg, 1982). Delays in reading or arithmetic in primary school may occur because of deficits in attention control and representational awareness or because of specific encoding difficulties for letters or quantities. Difficulties in learning advanced mathematics and science concepts may relate with delays in building multiple level hierarchical relations and commanding specific symbol systems that may stand for principle-level concepts (Susac et al., 2014; Demetriou, 2020b).

Supporting developmental priorities at the appropriate age requires appropriate screening systems that would diagnose children with difficulties in commanding the developmental priority of their age. In preschool, children must be assessed for attention control processes, and representational awareness and management processes. Children diagnosed with difficulties in any of these domains must receive special relevant support. For attention control, instruction must enable children to manage perceptual systems and action in reference to goals; for instance, alternate attention between stimulus properties according to goals; for representational awareness, instruction may highlight the perceptual access/knowledge bonds or awareness of grammatical/syntactical aspects of language and meaning variation (Demetriou et al., submitted). For representational management, support requires the explicit use of alternative symbol systems to symbolize concepts and actions, such as language, drawings, or photographs. This training must be combined with school-specific training. Children must command both the phonology of their language and the visual-phonological structure of a writing system (Demetriou et al., 2002; Kazi et al., 2019).

Children in early primary school must be assessed for difficulties in rule induction, relational processing, working memory management, and awareness of mental processes related to learning, such as linguistic, numerical, and concept formation related to learning to learn tasks in reading, arithmetic, and related fields. Diagnosis in this cycle must also identify problems in the awareness and relational integration processes which may have been left over from preschool and focus on the mental command of representational integration, as needed by inductive reasoning and working memory. Simple Raven-like tasks requiring integration over one or two dimensions of properties are appropriate (see Demetriou et al., submitted; Kazi et al., 2019). Children must be trained to process similarities and differences between objects vis-à-vis specific dimensions and explicitly conceptualize and specify the dimensions involved.

Also, learning must enhance awareness of representational units involved and of the role of cognitive processes in connecting them and holding them in memory, such as reasoning and rehearsal. In late primary school, children must be assessed for inferential and semantic flexibility in abstracting relations from implicit properties, as in multi-dimensional Raven-like matrices; they should also be assessed for abstracting meaning from texts and other sources of information, such as scripts stories and mathematical expressions, and integrating over sources. Remedial education should focus on training metaphorical, analogical, and deductive reasoning so that thinkers can access and systematically operate on the processes involved. Cognizance training should enable children to know their strengths and weaknesses in different processes and domains. In late primary school, education must prioritize reasoning and information management and the construction of self-organization and self-evaluation strategies. This applies especially to average ability students. Teachers must enable these students to understand the limitations of rule-based thought and be reflective on their successes and failures so that they may take compensatory action accordingly. Special programs must focus on learning skills facilitating depth of processing information and understanding at several levels in a text.

In adolescence, assessment must focus on reasoning, epistemic awareness, and precision in self-representation and self-evaluation. Adolescents with difficulties in commanding deductive reasoning must have the opportunity to grasp the notions and constraints of deductive reasoning along the lines described above. They must also be trained to understand the basic aspects of scientific reasoning (Kuhn, 2008). Therefore, in secondary school, education must enable adolescents to construct accurate self-representations about their cognitive and personality profile; this would enable them to embark on appropriate choices and acquire problem-solving strategies and interests tuned to their profile to maximize the output of their activity. Helping over-optimistic individuals to come down to earth may be helpful for their overall developmental prospects. Alternatively, it would be helpful for high ability students to know that their high ability is not always enough for high success at school; students must dedicate sustained effort and long-term organization fully capitalize on the ability and talent available. These changes are associated with education in problem solving and critical thinking.

This paper focused on the relations between cognition, development of cognitive competence and education. Obviously, learning at school has other important dimensions as well, including personality, motivation, and the cultural context of learning. These important aspects of learning at school at discussed elsewhere (Demetriou et al., 2022b).

## Data availability statement

The original contributions presented in the study are included in the article/supplementary material; further inquiries can be directed to the corresponding authors.

## Author contributions

AD, GS, SG, NM, RP, and SK contributed to conception and design of the manuscript. AD, GS, and SG wrote the first draft of the manuscript. NM, RP, and SK wrote sections of the manuscript. All authors contributed to the article and approved the submitted version.

## Conflict of interest

The authors declare that the research was conducted in the absence of any commercial or financial relationships that could be construed as a potential conflict of interest.

## Publisher’s note

All claims expressed in this article are solely those of the authors and do not necessarily represent those of their affiliated organizations, or those of the publisher, the editors and the reviewers. Any product that may be evaluated in this article, or claim that may be made by its manufacturer, is not guaranteed or endorsed by the publisher.
